# Special Issue: “James D. McChesney, Vision, Passion and Leadership in the Development of Plant-Derived Natural Products”

**DOI:** 10.3390/molecules26247415

**Published:** 2021-12-07

**Authors:** Larry A. Walker, N. P. Dhammika Nanayakkara

**Affiliations:** National Center for Natural Products Research, The University of Mississippi, University, MS 38677, USA; dhammika@olemiss.edu

It is a distinct pleasure for me to offer something in recognition of and tribute to Dr. James Dewey McChesney (Figure 1) in this special issue of Molecules. I have had the pleasure of knowing Jim for more than 40 years, ever since I arrived as a young faculty member at the University of Mississippi School of Pharmacy. We worked closely together until 1996, but even since that time, our collaborative relationship and close friendship have grown, and I have benefitted immensely from his mentoring, his scientific acumen, his vast experience, and his thought leadership in the natural products field. 

Jim was born near Hatfield, a small town in Missouri, in 1939 and completed a BSc in chemical technology from Iowa State University in 1961. In 1964, he received an MA in botany from the University of Indiana, and, in the following year, he received a Ph.D. in organic chemistry from the same institution. Soon after, he joined the Department of Botany and later the School of Pharmacy at the University of Kansas and became a Professor of Botany and Medicinal Chemistry. In 1978, he joined the Department of Pharmacognosy in the School of Pharmacy at The University of Mississippi as chair and in 1986 he became the Director of the Research Institute of Pharmaceutical Science. In 1996, he joined NaPro Biotherapeutics in Boulder, Colorado as the Vice President of Development. He became the chief scientific officer of Tapestry Pharmaceuticals and then ChromaDex of Boulder, Colorado in 2003. He moved back to his farm home near Etta, Mississippi in 2009 and founded his own companies (Ironstone Separations, Arbor Therapeutics, and Cloaked Therapeutics) in order to pursue his interest in the commercial development of natural products.

Dr. McChesney has done outstanding work throughout his long career in the development of plant-derived secondary metabolites as drug candidates, agrochemicals and food additives, as evidenced by his extensive publication record. Along the way, he has pioneered a number of methods for chromatographic separation, characterization, and analysis.

But beyond the discovery and analysis of phytochemicals, Jim has pioneered many impressive advances in the synthesis of important natural products, intermediates, analogs, derivatives, and metabolites. His accomplishments here feature a substantial body of work on the semi-synthesis of taxanes and their intermediates, which are the subject of numerous patents late in his career. These efforts led to the discovery and development of the third generation taxane analog TPI-287. This tubulin-binding and microtubule-stabilizing agent can also cross the blood-brain barrier, and has been advanced in a number of clinical trials for the treatment of several types of cancer and neurodegenerative diseases. In addition to his contributions to the preclinical and clinical development of taxanes, for more than 40 years he substantially advanced our understanding of the 8-aminoquinoline antimalarials, of which primaquine is the prototype. The 8-aminoquinolines are a critically important antimalarial drug class because of their unique activity on the dormant liver stages of the parasite. His efforts on 8-aminoquinoline chemistry, synthesis and metabolism led to important new insights; these efforts resulted in the development of NPC1161B, a drug candidate which is not only active against all stages of the life cycle of plasmodium in humans, but also highly effective in the treatment of leishmaniasis and *Pneumocystis* pneumonia. This compound is currently at the early clinical development stage.

But these are by no means the only examples. He developed an array of synthetic approaches, including a number of stereoselective routes, for artemisinins antimalarial drugs, cytokinins, hydrophenanthrenes, and several other natural-product or natural-product-inspired classes. 

Jim has also contributed immensely to overcome the inherent challenges in the development of nature-based products as potential drugs, agrochemicals and food additives. His work in this area focused on searching for plant varieties with a high content of active compounds—particularly in plant parts such as leaves, in order to harvest them in a sustainable manner—as well as micropropagating these strains in order to rapidly mass produce plants with uniform chemical profiles. His research on galanthamine and podophyllotoxin exemplify these efforts. He also led efforts to improve the production and processing of glycosides from *Stevia* for commercial markets. 

Dr. McChesney was chair of the Department of Pharmacognosy at Ole Miss until 1987, leading a small, but highly collaborative and productive group of natural products researchers. Early in his career, he had developed ties with scientists at several universities in Brazil, a relationship which continued throughout his career, notably including a Fulbright Fellowship in 1985 in Fortaleza. During this time and over the next decade as Director of the Research Institute of Pharmaceutical Sciences (RIPS), he was nurturing an ambitious vision for a national effort in the discovery and development of natural products. 

This was one of Jim’s signal career accomplishments: the conception and implementation of the National Center for Natural Products Research (NCNPR). Working with USDA scientists, university administrators and legislators, he devised a plan for this center, which was envisioned as a partnership between the state, the USDA, and the industry. In the early 1990s, the project began to take shape in earnest under Jim’s guidance. When the doors opened in 1995, it was truly a testament to his scientific leadership and vision, and the recognition of his acumen in government, political, and scientific circles.

Jim is known and respected as a hardnosed scientist. His mind is keenly tuned to absorbing and processing and thinking critically about research problems. The breadth and depth of his understanding always illuminate and challenge other researchers, and may sometimes even intimidate. On the other hand, those who come to know Jim find in him a true friend, a tender-hearted man, a gentleman in his own rugged way, a citizen of global awareness and allegiance with compassion for all humanity. 

Jim McChesney has been an eminent scholar and a tireless workman. He still is, even into his ninth decade. This special issue includes several articles that represent a small sampling of his research legacy in the form of contributions from his students, colleagues and co-workers through whom he continues in leading the unraveling of the wonders of the natural world. The issue focuses on the areas of natural-product drug discovery and dietary supplements with a special emphasis on the isolation of biologically active constituents, the determination of their mechanisms of action and structure activity relationships, and the development of analytical methods. Thirteen research papers and three reviews appear in this issue. 

In the first article, Li et al. [1] reported that the antiviral natural product, glycyrrhizic acid, prevents SARS-CoV-2 infection by complexing with the S-protein, including one mode of binding at the angiotensin-converting enzyme-receptor site, and thereby blocking viral entry into the host cells. 

The development of a UHPLC/Q-TOF analytical method to detect process contaminants 3-monochloro-propane-1,2-diol esters and glycidyl esters in glycerin was reported by Girad et al. [2]. Glycerin is widely used as a food additive and these contaminants have been identified as potential toxins, with the kidneys as their main target. 

Perera et al. [3] described the anti-inflammatory and antidiabetic properties of one new and six known curcubitane glycosides from *Momordica charantia* fruits, including in silico docking studies of these compounds with α-amylase and β-glucosidase. 

Kumarihamy et al. [4] described the bioassay-guided isolation and identification of secondary metabolites with antimalarial and phytotoxic activity from the endophytic fungus *Botryosphearia dothidea.*

Muhammad et al. [5] reported the identification of several antimicrobial constituents from *Machaerium* Pers. and their inhibitory activities and synergism against methicillin-resistant *Staphylococcus aureus*, vancomycin-resistant *Enterococcus faecium*, and permeabilized Gram-negative pathogens.

Zai et al. [6] described the isolation and identification of thermally induced products formed in the traditional way of thermally processing of rhizomes of the Chinese medicinal plant *Atractylodes macrocephala*. These compounds are reported not to be cytotoxic towards mammalian cancer and noncancer cell lines and are devoid of any activity against pathogenic micro-organisms. 

Chaurasiya et al. [7] reported the inhibition and kinetics of human monoamine oxidase A- and B-six by O-methylated flavonoids that were isolated from five different plants. The analysis of enzyme-inhibition kinetics was used to determine the mechanism of inhibition and molecular docking studies were used to suggest the possible binding site.

Xu et al. [8] described the bioassay-guided isolation and identification of dimeric naphtho-pyrones and other metabolites from the endophytic fungus *Teratosphaeria* sp. AK1128 and their cytotoxic activity. 

Radapong et al. [9] demonstrated that oxyresveratrol, the major constituent in the Thai medicinal plant *Artocapus lakoocha*, possesses pro-oxidant activity facilitated by the presence of copper, as well as significant DNA-damaging activity.

Ccana-Ccapatinta et al. [10] reported the identification of *Dalbergia ecastaphyllum* and *Symphonia globulifera* as the botanical sources of polyprenylated benzophenones in Brazilian red propolis by carrying out phytochemical and chromatographic analyses of plants that were collected by field surveys.

Rudnik et al. [11] reported that the cytotoxic activity of curcumin against non-small-cell lung cancer cells could be enhanced by formulating it as polymeric nanoparticles with or without methotrexate. 

Wang et al. [12] reported the characterization and quantification of oils from different parts of avocado fruits and used these results to assess the quality of avocado oils on the market as well as to suggest approaches to identify adulterated products.

Ali et al. [13] reported the insecticidal and biting deterrent activities of *Magnolia grandiflora* essential oils and selected pure compounds against the mosquito *Aedes aegypti.*

Three review papers also appeared in this issue. Perera and McChesney [14] reviewed the approaches to the separation, modification, identification, and scale-up purification of tetra-cyclic diterpene glycosides from *Stevia rebaudiana*. These glycosides are used as sweetening agents in the USA, Europe, and several other countries. 

The difficulty in procuring plant material in sufficient quantities is a major impediment for the development of natural-product-based pharmaceuticals and herbal medicines. Micropropagation techniques can be used to overcome this problem. Moraes et al. [15] reviewed the application of micropropagation techniques to mass produce medicinal plants and other important crops. They cited examples of how micropropagation techniques have successfully been used to generate phenotype lines that elicit the desired biological activity and to mass produce high-yielding chemotypes in order to isolate biologically active compounds on a commercial scale.

Tesfaye et al. [16] reviewed the anticancer compounds isolated from Ethiopian medicinal plants that are traditionally used in the treatment of cancer. This review included 119 papers appeared in the scientific literature between 1968 and 2020, and the structures of anticancer compounds that were isolated from 27 medicinal plants belonging to 18 different families. 

## Figures and Tables

**Figure 1:**
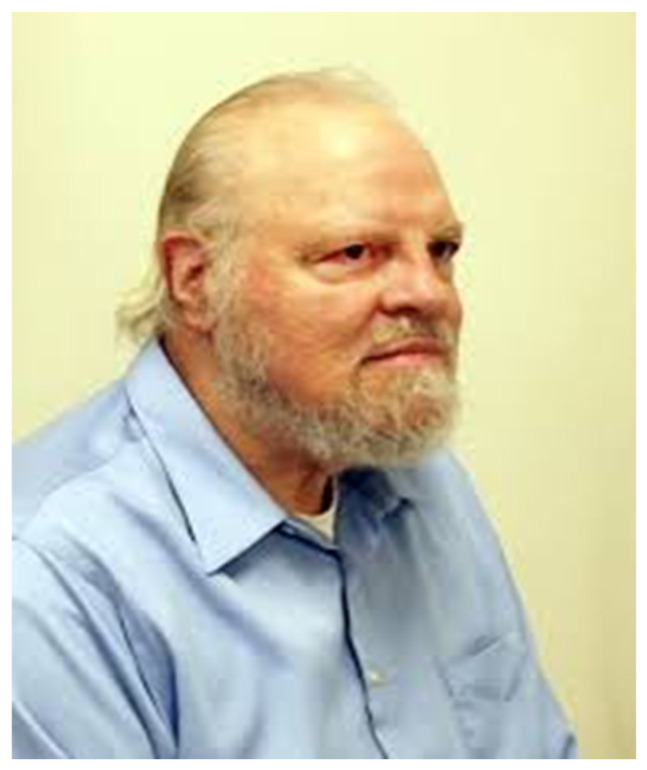
Dr. James D. McChesney.

## Data Availability

Not applicable.

## References

[1] Li J., Xu D., Wang L., Zhang M., Zhang G., Li E., He S. (2021). Glycyrrhizic Acid Inhibits SARS-CoV-2 Infection by Blocking Spike Protein-Mediated Cell Attachment. Molecules.

[2] Girard L., Herath K., Escobar H., Reimschuessel R., Ceric O., Jayasuriya H. (2021). Development of UHPLC/Q-TOF Analysis Method to Screen Glycerin for Direct Detection of Process Contaminants 3-Monochloropropane-1,2-diol Esters (3-MCPDEs) and Glycidyl Esters (GEs). Molecules.

[3] Perera W.H., Shivanagoudra S.R., Pérez J.L., Kim D.M., Sun Y., Jayaprakasha G.K., Patil B.S. (2021). Anti-Inflammatory, Antidiabetic Properties and In Silico Modeling of Cucurbitane-Type Triterpene Glycosides from Fruits of an Indian Cultivar of *Momordica charantia* L.. Molecules.

[4] Kumarihamy M., Rosa L.H., Techen N., Ferreira D., Croom E.M., Duke S.O., Tekwani B.L., Khan S., Nanayakkara N.P.D. (2021). Antimalarials and Phytotoxins from *Botryosphaeria dothidea* Identified from a Seed of Diseased *Torreya taxifolia*. Molecules.

[5] Muhammad I., Jacob M.R., Ibrahim M.A., Raman V., Kumarihamy M., Wang M., Al-Adhami T., Hind C., Clifford M., Martin B. (2020). Antimicrobial Constituents from *Machaerium* Pers.: Inhibitory Activities and Synergism of Machaeriols and Machaeridiols against Methicillin-Resistant *Staphylococcus aureus*, Vancomycin-Resistant *Enterococcus faecium*, and Permeabilized Gram-Negative Pathogens. Molecules.

[6] Zhai C., Zhao J., Chittiboyina A.G., Meng Y., Wang M., Khan I.A. (2020). Newly Generated Atractylon Derivatives in Processed Rhizomes of *Atractylodes macrocephala* Koidz. Molecules.

[7] Chaurasiya N.D., Midiwo J., Pandey P., Bwire R.N., Doerksen R.J., Muhammad I., Tekwani B.L. (2020). Selective Interactions of *O*-Methylated Flavonoid Natural Products with Human Monoamine Oxidase-A and -B. Molecules.

[8] Xu Y.-M., Arnold A.E., U′Ren J.M., Xuan L.-J., Wang W.-Q., Gunatilaka A.A.L. (2020). Teratopyrones A–C, Dimeric Naphtho-γ-Pyrones and Other Metabolites from *Teratosphaeria* sp. AK1128, a Fungal Endophyte of *Equisetum arvense*. Molecules.

[9] Radapong S., Sarker S.D., Ritchie K.J. (2020). Oxyresveratrol Possesses DNA Damaging Activity. Molecules.

[10] Ccana-Ccapatinta G.V., Mejía J.A.A., Tanimoto M.H., Groppo M., Carvalho J.C.A.S.d., Bastos J.K. (2020). *Dalbergia ecastaphyllum* (L.) Taub. and *Symphonia globulifera* L.f.: The Botanical Sources of Isoflavonoids and Benzophenones in Brazilian Red Propolis. Molecules.

[11] Rudnik L.A.C., Farago P.V., Manfron Budel J., Lyra A., Barboza F.M., Klein T., Kanunfre C.C., Nadal J.M., Bandéca M.C., Raman V. (2020). Co-Loaded Curcumin and Methotrexate Nanocapsules Enhance Cytotoxicity against Non-Small-Cell Lung Cancer Cells. Molecules.

[12] Wang M., Yu P., Chittiboyina A.G., Chen D., Zhao J., Avula B., Wang Y.-H., Khan I.A. (2020). Characterization, Quantification and Quality Assessment of Avocado (*Persea americana* Mill.) Oils. Molecules.

[13] Ali A., Tabanca N., Demirci B., Raman V., Budel J.M., Baser K.H.C., Khan I.A. (2020). Insecticidal and Biting Deterrent Activities of *Magnolia grandiflora* Essential Oils and Selected Pure Compounds against *Aedes aegypti*. Molecules.

[14] Perera W.H., McChesney J.D. (2021). Approaches toward the Separation, Modification, Identification and Scale up Purification of Tetracyclic Diterpene Glycosides from *Stevia rebaudiana* (Bertoni) Bertoni. Molecules.

[15] Moraes R.M., Cerdeira A.L., Lourenço M.V. (2021). Using Micropropagation to Develop Medicinal Plants into Crops. Molecules.

[16] Tesfaye S., Asres K., Lulekal E., Alebachew Y., Tewelde E., Kumarihamy M., Muhammad I. (2020). Ethiopian Medicinal Plants Traditionally Used for the Treatment of Cancer, Part 2: A Review on Cytotoxic, Antiproliferative, and Antitumor Phytochemicals, and Future Perspective. Molecules.

